# Serum Lactate as Serum Biomarker for Cardiopulmonary Parameters within the First 24 Hours after a Spontaneous Intracerebral Hemorrhage

**DOI:** 10.3390/diagnostics12102414

**Published:** 2022-10-05

**Authors:** Michael Bender, Kristin Haferkorn, Jasmin Nagl, Eberhard Uhl, Marco Stein

**Affiliations:** Department of Neurosurgery, Justus-Liebig-University Gießen, 35392 Giessen, Germany

**Keywords:** serum lactate, intracerebral hemorrhage, NICU, serum biomarker

## Abstract

Objective: Cardiopulmonary (CP) complications are well known in patients with an intracerebral hemorrhage (ICH) and could be associated with a higher serum lactate level. The present study aimed to assess the associations between the initial serum lactate level and the CP parameters within the first 24 h of intensive care unit (ICU) treatment in neurosurgical ICH patients. Patients and Methods: A total of 354 patients admitted to the ICU between 01/2009 and 12/2017 with a diagnosis of an ICH were retrospectively analyzed. Blood samples were taken upon admission, and each patient’s demographic, medical, and radiological data upon admission, as well as several CP parameters, were recorded within the first 24 h of ICU treatment. Results: A higher serum lactate level was associated with a lower GCS score (*p* < 0.0001), as well as a higher Acute Physiology and Chronic Health Evaluation II score (*p* = 0.002) upon admission. Additionally, patients with initially higher serum lactate levels had a significantly higher need for a norepinephrine application (*p* = 0.004) and inspiratory oxygen fraction (*p* = 0.03) within the first 24 h. Conclusion: Neurosurgical ICH patients with higher serum lactate levels upon admission require more CP support within the first 24 h of ICU treatment.

## 1. Introduction

A spontaneous intracerebral hemorrhage (ICH) is still associated with a high rate of morbidity and mortality, and only a fifth of all patients recover to live an independent life [1,2,3,4,5,6,7,8]. In particular, the neurological deterioration occurs in up to 30% of ICH patients within the first 24 h due to the expansion of the ICH [6,7,8]. Therefore, this period of ICU treatment is important concerning patients’ outcomes [8]. An essential part of the intensive care unit (ICU) treatment for ICH patients is avoiding a secondary cardiopulmonary (CP) complication, which could lead to tissue hypoperfusion and hypoxemia and may result in an additional cerebral injury. However, CP complications, including electrocardiographic and cardiac wall motion abnormalities, as well as pulmonary alterations, are often seen in ICH patients [9,10,11,12,13]. The pathophysiological pathway of these complications could be justified by a neurogenic triggered autonomic dysregulation with an excessive catecholamine response to the circulation [1,2,6,10]. Nevertheless, the exact mechanism remains unclear.

A higher serum lactate level upon admission is a well-known and commonly used parameter to determine the illness severity and the prediction of the outcome in various diseases, including sepsis and septic shock, cardiac arrest, trauma, regional ischemia, and liver dysfunction [14,15,16,17,18,19,20]. The elevation of serum lactate levels is frequently caused by an increased production (e.g., hypoperfusion due to the circulation dysfunction or anaerobic metabolic conditions within hypoxia) and/or a decreased elimination (e.g., liver dysfunction) and could reflect systemic cardiopulmonary complications [14]. Previous studies reported on the negative influence of the initially raised serum lactate level on the outcome and mortality in ICH patients [6,21]. However, the association between the initially higher serum lactate level and CP complications within the first 24 h of ICU treatment in neurosurgical ICH patients is still unknown. Early identification of ICH patients with a potentially higher need for CP support could be helpful for improving ICU treatment, especially regarding the timely implementation of more invasive pulmonary and circulation monitoring. Therefore, the present study was conducted to investigate the impact of the serum lactate levels upon admission, as an early serum biomarker, on the CP parameters within the first 24 h after a spontaneous ICH in neurosurgical ICU patients.

## 2. Materials and Methods

### 2.1. Study Design and Population

**Figure 1:**
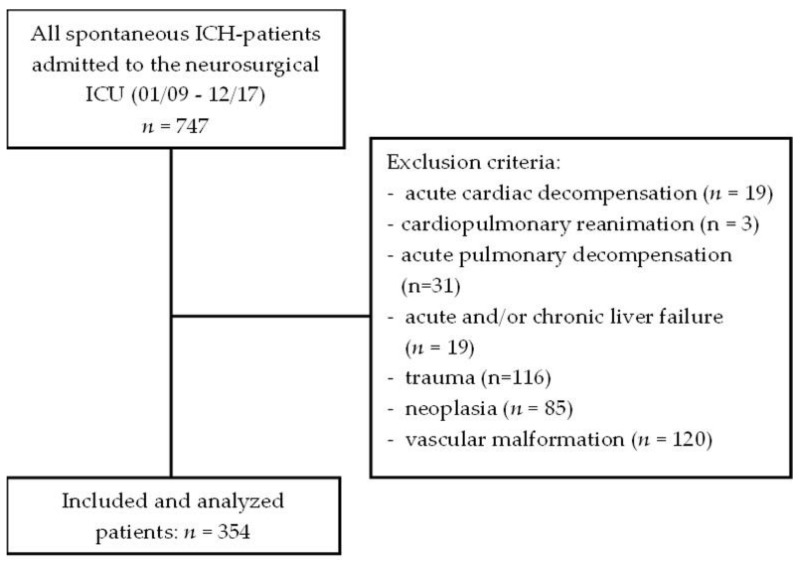
Flowchart for the inclusion and exclusion criteria.

The current study is a retrospective analysis of all patients with a spontaneous ICH aged above 18 years who were treated for at least 24 h at the neurosurgical ICU of the University Hospital Giessen from January 2009 to December 2017 (*n* = 747). The study protocol was approved by the ethical committee of Justus-Liebig-University (No: 95/17). The diagnosis of an ICH was confirmed by a computed tomography (CT) scan upon admission. The exclusion criteria were acute cardiac decompensation (*n* = 19), cardiopulmonary reanimation (*n* = 3), acute pulmonary decompensation (*n* = 31), acute and/or chronic liver failure (*n* = 19), and/or ICH due to trauma (*n* = 116), neoplasia (*n* = 85), or vascular malformation (*n* = 120), resulting in a total number of 354 included patients, as shown in Figure 1.

The patients’ baseline data, comorbidities and premedication, CP, serum biomarkers, intensive care unit management and treatment regimes, radiological data, and intrahospital outcomes were extracted from the medical records and analyzed.

### 2.2. Baseline Data

The baseline data contain demographic data, the Glasgow Coma Scale (GCS) score, the body mass index (BMI), and the Acute Physiology and Chronic Health Evaluation II (APACHE II) score upon admission, as well as during the overall hospital stay [22,23].

### 2.3. Comorbidities and Premedication

The medical records were analyzed with regard to comorbidities and premedication. Comorbidities were defined as preexisting chronic arterial hypertension, chronic obstructive pulmonary diseases, cardiac arrhythmia, coronary artery disease, heart failure, chronic renal insufficiency, and/or diabetes mellitus. Furthermore, premedication was stratified into long-term medication with antihypertensive drugs, antiobstructive drugs, antidiabetic drugs, antiplatelet agents, new oral anticoagulants, and/or vitamin K antagonists.

### 2.4. Cardiopulmonary Parameters

The average inspiratory oxygen fraction (FiO2), the average norepinephrine application rate (NAR), and the necessity of endotracheal intubation within the first 24 h were defined and analyzed as CP parameters. In addition, the mean arterial blood pressure, heart rate, arterial oxygen, partial pressure, and positive end expiratory pressure (PEEP) within the first 24 h of ICU treatment, as well as the body temperature upon admission, were examined. All CP parameters were recorded continuously at 5-min intervals and stored in the digital ICU data recording system. These data were used for the study analyses.

### 2.5. Serum Biomarker

Blood samples were immediately drawn upon the patients’ admission to the neurosurgical department. Serum lactate levels in mmol/L (ADVIA Chemistry XPT^®^ LAC Assay, Siemens, Germany) were defined as serum biomarkers for CP parameters. In addition, white blood cell count in giga/L (XE 5000 Hematology Analyzer, Sysmex, Germany), hemoglobin level in g/dL (XE 5000 Hematology Analyzer, Sysmex, Germany), hematocrit level in L/L (XE 5000 Hematology Analyzer, Sysmex, Germany), cholinesterase in U/L (ADVIA Chemistry XPT^®^, Siemens, Germany), blood glucose level in mg/dL (ADVIA Chemistry XPT^®^, Siemens, Germany), serum lactate level in mmol/L (ADVIA Chemistry XPT^®^, Siemens, Germany), troponin I in µg/dL (ADVIA Centaur XPT^®^, Siemens, Germany), cortisol level in µg/dL (ADVIA Centaur XPT^®^, Siemens, Germany), C-reactive protein (CRP) in mg/L (ADVIA Chemistry XPT^®^, Siemens, Germany), albumin level in g/L (ADVIA Chemistry XPT^®^, Siemens, Germany), creatine in mg/dL (ADVIA Chemistry XPT Crea assay; Siemens, Germany), prothrombin time in % (Atellica^®^ COAG 360 System, Siemens, Germany), partial thromboplastin time in sec (Atellica^®^ COAG 360 System, Siemens, Germany), antithrombin III in %/NORM (Atellica^®^ COAG 360 System, Siemens, Germany), and fibrinogen in g/L (Atellica^®^ COAG 360 System, Siemens, Germany) upon admission were recorded and analyzed in all of the included patients.

### 2.6. Treatment Regimen and Intensive Care Unit Management

All patients were initially admitted to the emergency department of our university hospital. Following the confirmation of the diagnosis of an ICH, patients were treated directly or immediately after surgical treatment in our neurosurgical ICU for at least 24 h. According to the clinical and/or radiological conditions of the patients, an indication for medical or additional surgical treatment was performed by a neurosurgeon consultant. The medical treatments comprise all options for conservative ICU treatments. Therefore, the targets of the cardiopulmonary parameters were defined as a systolic arterial blood pressure between 120 and 140 mmHg and an arterial oxygen partial pressure ≥ 100 mmHg, independent of a surgical or conservative procedure. In all ICU patients, cardiopulmonary monitoring, including an invasive blood pressure measurement catheter (Combitrans Monitoring Set arteriell; B. Braun, Melsungen, Germany), a pulse oximeter (Nellcor adult SpO2 sensor; Covidien LLC, Mansfield, MA, USA), and a 3-lead electrocardiogram (B. Braun, Melsungen, Germany), was carried out. For the application of intravenous drugs, a central venous catheter (Arrow International, Inc., Reading, PA, USA) was implemented in all ICH patients. Furthermore, arterial blood samples were taken every four hours for blood gas analysis (ABL800 FLEX; Radiometer, Copenhagen Denmark and Krefeld, Germany). Endotracheal intubation and mechanical ventilation were performed (Servo-I; Maquet, Rastatt, Germany) in cases of respiratory insufficiency or a GCS score of less than nine. Continuous analgosedation was performed using midazolam (5–40 mg/h) or propofol (200–500 mg/h) in combination with sufentanil (35–100 µg/h). Additional surgical treatments included the insertion of an external ventricular drain (EVD), the evacuation of the ICH, decompressive craniectomy, or decompressive craniectomy with evacuation of the ICH.

### 2.7. Radiological Data

The initial CT scans of all of the included patients were evaluated concerning the localization of the ICH (lobar supratentorial, deep supratentorial, and infratentorial) and the presence of an IVH (Graeb score > 1) and/or hydrocephalus (Evans’ Index > 0.3) by two independent individuals (i.e., K.H. and M.B.) [24,25]. Furthermore, the formula A × B × C/2 was used to calculate the volume.

### 2.8. Intra-Hospital Outcome and Mortality

The modified Rankin Scale (mRS) at discharge was used to evaluate the intra-hospital outcome and mortality [26]. For the evaluation of the functional outcome, the ICH patients were stratified into patients with favorable (mRS 0–4) and those with unfavorable (mRS 5–6) outcomes.

### 2.9. Statistical Analysis

The data analysis was performed using the Statistical Package for the Social Sciences version 15.0 for Windows (Version 15.0; SPSS Inc., Chicago, IL, USA) and Prism Version 5 statistical software (GraphPad Software, Inc., La Jolla, CA, USA). To analyze the impact of the initial serum lactate level on the CP parameters within the first 24 h, patients were stratified into patients with a serum lactate level ≤ 1.72 mmol/L (lactate negative) and patients with a serum lactate level > 1.72 mmol/L (lactate positive). The cut-off level was calculated using the average serum lactate level of the entire study population (1.7 mmol/L ± 1.5 mmol/L). Data were expressed as the median and interquartile range (IQR) for non-normal distributions and the mean ± standard deviation for patients with normally distributed parameters. The Mann–Whitney U test or Student’s *t* test were used for the univariate analysis, and the Chi-square test was used to identify the differences in binary variables in both groups. A *p*-value of <0.05 was defined as the level of significance.

## 3. Results

### 3.1. Main Characteristics

The entire study population consisted of 354 patients (161 women and 193 men) with a mean age of 68.6 ± 13 years. Elevated serum lactate levels upon admission were found in 115 (32.5%) patients. Overall, a median GCS score of 8 (IQR 3–12) and an APACHE II score of 14 (IQR 11–19) were observed. The median length of inpatient treatment was 16.5 days (IQR 4.8–27). As for comorbidities, chronic arterial hypertension (58.8%) was most commonly found, followed by preexisting cardiac arrhythmia (19.8%). Furthermore, antihypertensive drugs were the most frequent premedications. The total study population required an average NAR of 0.03 ± 0.04 µg/kg/min and FiO2 of 34.6 ± 13.4 to achieve the CP targets within the first 24 h. Endotracheal intubation and mechanical ventilation were indicated in 211 patients (59.6%) within the first 24 h. Regarding the further cardiopulmonary parameters, a median heart rate/min of 75 (IQR = 64–87), median systolic blood pressure of 137 mmHg (IQR = 129–145.3), median PEEP level of 7 (IQR:6–9), arterial oxygen partial pressure of 109 mmHg (IQR = 98–123) were recorded within the first 24 h, and a body temperature of 36.3 (IQR: 35.5–36.9) was observed upon admission. Medical treatment was performed in 151 (42.7%) patients, while 203 (57.3%) required additional surgical treatment. The mean ICH volume at the initial CT scan was 52.3 cm^3^ ± 42.2 cm^3^, and deep supratentorial was the most commonly identified localization (50.8%). At discharge, a median mRS score of 5 (IQR = 4–6) was found, and 59.6% had an unfavorable outcome. Furthermore, intrahospital mortality was 31.4%. The main characteristics of the entire study population are summarized in Table 1.

### 3.2. Serum Lactate

**Figure 2:**
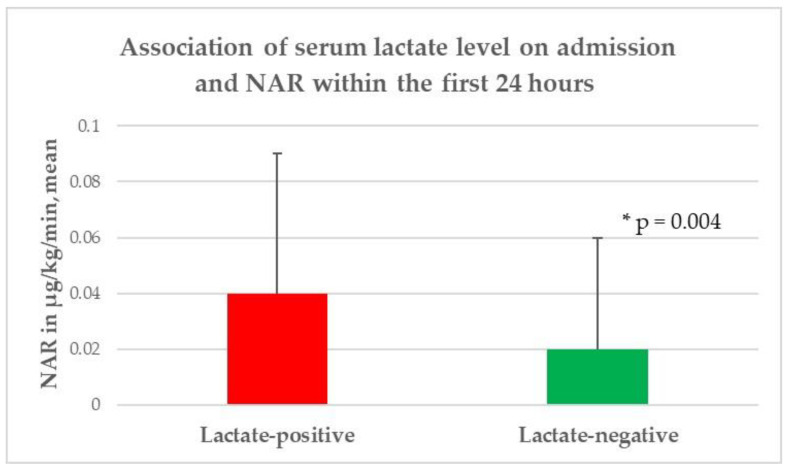
Association of the serum lactate level upon admission and NAR within the first 24 h. NAR: norepinephrine application rate, * level of significance.

**Figure 3:**
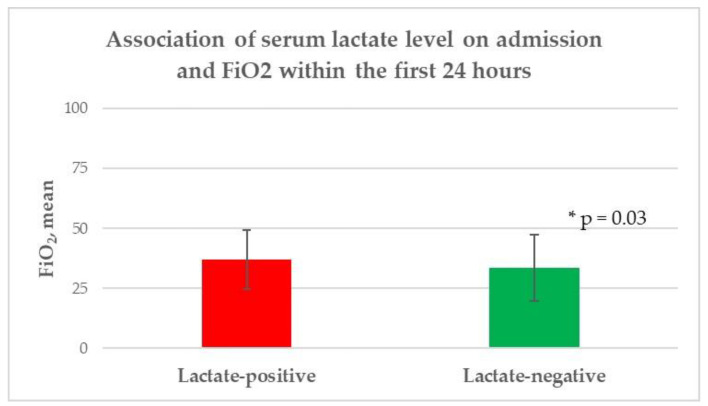
Association of the serum lactate level upon admission and FiO2 within the first 24 h. FiO_2_: inspiratory oxygen fraction, * level of significance.

**Figure 4:**
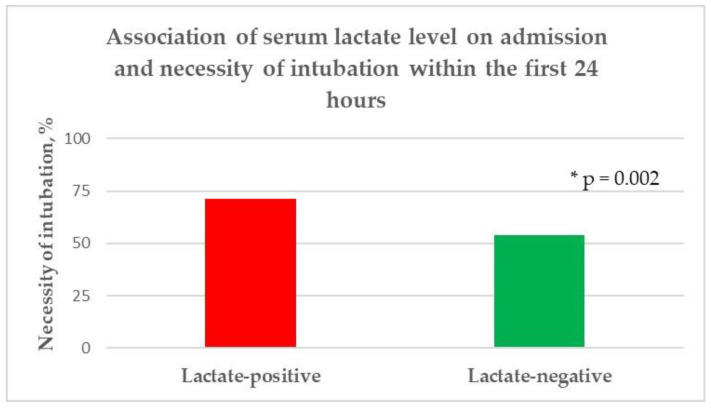
Association of the serum lactate level upon admission and necessity of endotracheal intubation within the first 24 h, * level of significance.

In the univariate analysis, no significant differences were identified between the patients with initially higher serum lactate levels and those with lower lactate levels with respect to age (*p* = 0.26), gender (*p* = 0.23), body mass index (*p* = 0.6), and length of hospital stay (*p* = 0.78). According to comorbidities and premedication, a significantly lower rate of chronic arterial hypertension (*p* = 0.02) and use of antihypertensive drugs (*p* = 0.0005) were observed in the lactate-positive group, while no further significant difference between the groups was discovered. The patients with higher serum lactate levels had higher white blood cell counts (*p* = 0.001), hemoglobin levels (*p* = 0.003), hematocrit levels (*p* = 0.02), blood glucose levels (*p* < 0.0001), and albumin levels upon admission. Additionally, the lactate-positive group required a significantly higher NAR (*p* = 0.004) and FiO2 (*p* = 0.03), as well as the necessity of endotracheal intubation (*p* = 0.002) to achieve the CP target within the first 24 h of ICU treatment, compared to the lactate-negative group (Figure 2, Figure 3 and Figure 4). In contrast, no significant difference was observed with regard to the mean arterial blood pressure values (*p* = 0.66), median heart rate/min values (*p* = 0.86), median arterial oxygen partial pressures (*p* = 0.13), and median PEEP levels (*p* = 0.71) within the first 24 h of ICU treatment; similarly, no significant difference was found in body temperature upon admission (*p* = 0.33) between the two groups. Moreover, no significant difference was found with respect to the treatment regime (*p* = 0.11), ICH volume (*p* = 0.11), presence of IVH (*p* = 0.4), and hydrocephalus (*p* = 0.29), as well as the lobar supratentorial (*p* = 0.53), deep supratentorial (*p* = 0.57), and infratentorial localization (*p* = 0.1) of ICH between patients with higher serum lactate levels upon admission and patients with an initial lower serum lactate level. In addition, no significant difference was observed in the median mRS (*p* = 0.06), functional outcome (*p* = 0.3), and mortality (*p* = 0.52) between the lactate-positive and lactate-negative groups, as presented in Table 2.

## 4. Discussion

### 4.1. Summary of the Findings

A retrospective study of 354 neurosurgical ICH patients was performed to assess the association between initially higher serum lactate levels upon admission and CP parameters within the first 24 h of ICU treatment. To our knowledge, no comparable studies are currently available. In the present study, ICH patients with initially higher serum lactate levels had lower GCS scores and lower rates of preexisting chronic atrial hypertension and preexisting antihypertensive medication upon admission. They also had initially higher APACHE II scores, white blood cell counts, blood glucose levels, albumin levels, and hemoglobin and hematocrit levels. In addition, ICH patients with higher serum lactate levels upon admission required significantly more NAR, FiO_2_, and endotracheal intubation within the first 24 h of ICU treatment to reach the cardiopulmonary targets. Therefore, the initial serum lactate level has the potential to be a helpful early serum biomarker for improving ICU treatment, especially for the early detection of patients who could potentially develop CP complications within the first 24 h of ICU treatment.

### 4.2. Elevated Serum Lactate Level

The serum lactate level is an easily determined biomarker. The average serum lactate level in the current study was 1.7 mmol/L, which is comparable to those of previous studies [6,21]. In general, lactate is an important parameter in the human catabolic metabolism [14]. It is produced in almost all human tissues, most commonly in the muscles. The elimination of lactate occurs primarily by the liver and to a lesser extent by the kidneys [27,28,29]. In aerobic metabolism, pyruvate is the result of glycolysis followed by passing the Krebs cycle without a relevant production of lactate. In contrast, lactate is the end product of glycolysis under anaerobic conditions, subsequently entering the Cori cycle as a metabolite for gluconeogenesis [27,28,29].

In the present study, a higher serum lactate level was associated with a lower GCS score, lower preexisting chronic arterial hypertension and antihypertensive medication, as well as a higher APACHE II score and white blood cell count upon admission. The association of an initially lower GCS and higher APACHE II scores in the lactate-positive group is not surprising. Behrouz et al. reported an association of leukocytosis and a lower GCS upon admission in ICH patients, so that in the current study the lower GCS score as well as higher APACHE II score and white blood cell count upon admission may express a higher medical and neurological severity of the ICH patients in the lactate-positive group [30]. Furthermore, our findings revealed that patients with higher serum lactate levels had a lower rate of preexisting chronic arterial hypertension and antihypertensive medication upon admission. Elevation of serum lactate levels is frequently caused by a raised production (e.g., hypoxemia, hypoperfusion due to macro- and/or microcirculatory dysfunction, hypermetabolic conditions) and/or a reduced elimination (e.g., liver dysfunction) [14]. As an indicator of oxidative stress and hypoperfusion, the lower rate of raised serum lactate levels in patients with preexisting chronic arterial hypertension could be explained by a chronic adaptation of a disturbed microcirculation and catabolism in those patients [31]. Moreover, the impact of the initially higher serum lactate levels upon admission on outcome and mortality in ICH patients is scarcely described and controversial [6,8,21,32]. Similar to our results, no association between a higher serum lactate level upon admission and intra-hospital mortality was identified in a previous study [8]. In contrast, Oh et al. reported that elevated lactate levels were related to an increased overall 90-day mortality among a heterogeneous group of neuro-intensive care patients [32]. Additionally, Lehman et al. revealed an association of initially raised lactate levels after a spontaneous deep-seated intracranial hemorrhage with a poor outcome and mortality [21]. These controversial findings could be explained by the substantial inhomogeneous study populations as well as the limited amount of available studies.

### 4.3. Cardiopulmonary Parameters

The present study revealed that patients with higher serum lactate levels upon admission had initially higher levels of blood glucose and albumin as well as hemoglobin and hematocrit. Furthermore, these patients required more NAR, FiO_2_, and endotracheal intubation within the first 24 h to achieve the cardiopulmonary targets of ICU treatment. This may be explained by cardiac and pulmonary complications, which are frequently reported in ICH patients [9,10,11,12,13]. The pathophysiological pathway could be caused by an autonomic dysregulation, including an excessive catecholamine response to the systemic circulation [1,2,6,10]. The excessive release of catecholamine in combination with an increased intracranial pressure after an ICH could lead to a myocardial injury, including electrocardiographic abnormalities and wall motion dysfunction, even in the absence of coronary stenosis [6,9,10,11,12]. In addition, vasoconstrictive-related tissue hypoperfusion, tissue hypoxia, due to pulmonary edema, and increased metabolism may be the results of a neurogenic-mediated autonomic dysregulation, resulting in a higher serum lactate level and systemic cardiopulmonary decompensation. This pathophysiological pathway could explain the higher serum blood glucose level as well as the higher need for NAR, FiO_2_, and endotracheal intubation within the first 24 h in the lactate-positive group [21,33,34]. Additionally, the higher serum levels of albumin, hemoglobin, and hematocrit upon admission in patients with initially elevated serum lactate levels could indicate acute dehydration triggered by renal hyperperfusion due to an excessive release of catecholamine after ICH [33]. Regardless of the lower GCS score and higher APACHE II scores in the lactate-positive group, the mean arterial blood pressure, median heart rate, median arterial oxygen partial pressure, and median PEEP level were not significantly different in all of the patients within the first 24 h of ICU treatment, so that, in our opinion, both groups were comparable concerning NAR, FiO_2_, and the necessity of endotracheal intubation.

These findings emphasize that an initially higher serum lactate level upon admission could be a useful serum biomarker to predict a potentially higher need for CP support within the first 24 h of ICU treatment. Furthermore, patients with initially increased serum lactate levels should be continuously monitored, and they could probably benefit from early intensive and invasive hemodynamic monitoring, for example, by using a Swan-Ganz catheter and/or pulse contour cardiac output system (PiCCO) and/or intermittent or continuous transthoracic/transesophageal echocardiography [35].

### 4.4. Limitations and Strengths of the Study

The current study revealed some strengths but also several limitations. On this account, the results of the study should be interpreted with caution. First, there is the monocentric and retrospective character of the study. Secondarily, only a limited number of CP parameters within the first 24 h of ICU treatment were assessed. Evaluation of additional cardiac and pulmonary parameters (e.g., the global end-diastolic volume index, cardiac index, pulmonary vascular permeability index, and extravascular lung water) could be performed using a PiCCO system; however, it has its major use in septic patients [35]. In addition, serial serum lactate measurements were not available due to the retrospective design of the study. Moreover, in all ICU-admitted patients with an ICH, a 3-lead electrocardiography was performed and continuously monitored. Unfortunately, these were not digitally recorded, so a serial analysis was not feasible. Finally, the findings cannot be applied to all types of ICHs due to the exclusion of patients with trauma, malignancy, and/or vascular malformations. In addition, due to the low median GCS score and high mean ICH volume upon admission in the lactate positive group, we cannot investigate the association of initially higher lactate level and the need of cardiopulmonary support in ICH patients with a higher GCS score (e.g., >10) and smaller ICH volume (e.g., <20 mL or <30 mL). Nevertheless, this important aspect should be investigated in a further study.

The strength of the present study was the large study population with comprehensive demographic, laboratory chemistry, clinical, and radiological records of ICU-admitted neurosurgical patients with an ICH. In addition, to our knowledge, this is the first report evaluating the impact of serum lactate levels on the CP parameters within the first 24 h of ICU treatment in neurosurgical ICH patients.

Apart from the limitations, the findings of the current study could be helpful for improving ICU treatment within the first 24 h with respect to the early identification of patients with the potential of developing CP complications. These ICH patients may potentially benefit from more intensive and invasive hemodynamic monitoring.

## 5. Conclusions

The current study revealed for the first time the impact of the higher serum lactate levels upon admission on CP parameters within the first 24 h of ICU treatment in ICH patients. Neurosurgical ICU-admitted patients with an ICH and initially raised serum lactate levels had lower GCS and higher APACHE II scores upon admission. Additionally, a higher need for NAR, FiO2, and endotracheal intubation was required within the first 24 h in patients with initially increased serum lactate levels to achieve CP targets. Therefore, serum lactate has the potential to be an appropriate serum biomarker for improving ICU treatment within the first 24 h in neurosurgical ICH patients. Nevertheless, these results should be confirmed in a further prospective study.

## Figures and Tables

**Table 1:** Main characteristics of the entire study population (*n* = 354).

Parameter	Results
**Baseline data**	
Age, years, mean (±SD) *	68.6 (13)
Women, *n* (%) *	161 (45.5)
Men, *n* (%) *	193 (54.5)
Body Mass Index, kg/m^2^, median (IQR) *	26.1 (24.2–29.3)
Glasgow Coma Scale score, median (IQR) *	8 (3–12)
APACHE II score, median (IQR) *	14 (11–19)
Hospital stay, median (IQR) ***	16.5 (4.8–27)
**Comorbidities**	
Chronic arterial hypertension, *n* (%) *	208 (58.8)
Chronic obstructive pulmonary diseases, *n* (%) *	15 (4.2)
Cardiac arrhythmia, *n* (%) *	70 (19.8)
Coronary artery disease, *n* (%) *	44 (12.4)
Heart failure, *n* (%) *	20 (5.6)
Chronic renal insufficiency, *n* (%) *	21 (5.9)
Diabetes mellitus, *n* (%) *	56 (15.8)
**Premedication**	
Antihypertensive drugs, *n* (%) *	164 (46.3)
Antiobstructive drugs, *n* (%) *	5 (1.4)
Antidiabetic drugs, *n* (%) *	36 (10.2)
Antiplatelet agents, *n* (%) *	50 (14.1)
New oral anticoagulants, *n* (%) *	13 (3.7)
Vitamin K antagonist, *n* (%) *	73 (20.6)
**Cardiopulmonary parameters**	
Norepinephrine application rate, µg/kg/min, mean (±SD) **	0.03 (0.04)
Requiring norepinephrine, *n* (%) **	153 (43.2)
Systolic blood pressure, mmHg, median (IQR) **	137 (129–145.3)
Heart rate, beats per minute, median (IQR) **	75 (64–87)
Inspiratory oxygen fraction, mean (±SD) **	34.8 (13.4)
Endotracheal intubation, *n* (%) **	211 (59.6)
PEEP level, median (IQR) **	7 (6–9)
Arterial oxygen partial pressure (mmHg), median (IQR) **	109 (98–123)
Body temperature, centigrade, median (IQR) *	36.3 (35.5–36.9)
**Serum biomarkers**	
White blood cells, giga/L, mean (±SD) *	10.9 (4.4)
Hemoglobin, g/dL, mean (±SD) *	13.1 (2)
Hematocrit, l/L, mean (±SD) *	0.39 (0.05)
Cholinesterase, U/L, mean (±SD) *	7809.4 (2282.3)
Blood glucose, mg/dL, mean (±SD) *	163 (59.3)
Serum lactate, mmol/L, mean (±SD) *	1.7 (1.5)
Troponin I (*n* = 177), µg/dL, mean (±SD) *	0.31 (2.6)
Elevated Troponin I, *n* (%) *^,a^	49 (27.7)
Cortisol, µg/dL, mean (±SD) *	27.7 (19.4)
C-reactive protein, mg/L, mean (±SD) *	22 (39.9)
Albumin, g/L, mean (±SD) *	38.2 (5.4)
Creatinine, mg/dL, mean (±SD) *	0.9 (0.6)
Prothrombin time, %, mean (±SD) *	83.7 (26.2)
Partial thromboplastin time, seconds, mean (±SD) *	32.5 (11.4)
Antithrombin III, %/NORM, mean (±SD) *	88.7 (15.9)
Fibrinogen, g/L, mean (±SD) *	3.3 (1.1)
**Treatment regime**	
Medical treatment, *n* (%) ***	151 (42.7)
Additional Surgical Treatment, *n* (%) ***	203 (57.3)
**Radiological data**	
Localization	
Supratentorial, lobar, *n* (%) *	122 (34.4)
Supratentorial, deep, *n* (%) *	180 (50.8)
Infratentorial, *n* (%) *	52 (14.7)
ICH volume, cm^3^, mean (±SD)	52.3 (42.2)
IVH, *n* (%) *	248 (70.1)
Hydrocephalus, *n* (%) *	158 (44.6)
**Intra-hospital outcome**	
mRS score, median (IQR) ****	5 (4–6)
mRS 0, *n* (%) ****	0
mRS 1, *n* (%) ****	21 (5.9)
mRS 2, *n* (%) ****	24 (6.8)
mRS 3, *n* (%) ****	25 (7.1)
mRS 4, *n* (%) ****	73 (20.6)
mRS 5, *n* (%) ****	100 (28.2)
mRS 6, *n* (%) ****	111 (31.4)
Favorable outcome, *n* (%) ****	143 (40.4)
Unfavorable outcome, *n* (%) ****	211 (59.6)

SD: standard deviation, IQR: interquartile range, APACHE II: Acute Physiology and Chronic Health Evaluation II, PEEP: positive end expiratory pressure, ICH: intracerebral hemorrhage, IVH: intraventricular hemorrhage, mRS: modified Rankin Scale. * upon admission, ** within the first 24 h, *** during inpatient treatment, **** at discharge, ^a^ TNI value > 0.05 µg/L was defined as elevated according to the local laboratory parameters.

**Table 2:** Univariate analysis of the serum lactate level.

Parameter	Lactate-Positive (*n* = 115)	Lactate-Negative (*n* = 239)	*p*-Value
**Baseline Data**			
Age, years, mean (±SD) *	67.4 (12.5)	69.1 (13.4)	0.26
Women, *n* (%) *	47 (40.9)	114 (47.7)	0.23
Men, *n* (%) *	68 (59.1)	125 (52.3)	
Body Mass Index, kg/m^2^, median (IQR) *	26.1 (24.2–29.4)	26.1 (23.9–28.7)	0.6
Glasgow Coma Scale score, median (IQR) *	7 (3–10)	9 (4–13)	<0.0001
APACHE II score, median (IQR) *	16 (13–19)	14 (10–18)	0.002
Hospital stay, median (IQR) ***	18 (4–29)	19 (5–26)	0.78
**Comorbidities**			
Chronic arterial hypertension, *n* (%) *	57 (49.6)	151 (63.2)	0.02
Chronic obstructive pulmonary diseases, *n* (%) *	3 (2.6)	12 (5)	0.29
Cardiac arrhythmia, *n* (%) *	20 (17.4)	50 (20.9)	0.44
Coronary artery disease, *n* (%) *	12 (10.4)	32 (13.4	0.43
Heart failure, *n* (%) *	9 (7.8)	11 (4.6)	0.22
Chronic renal insufficiency, *n* (%) *	5 (4.3)	16 (6.7)	0.38
Diabetes mellitus, *n* (%) *	22 (19.1)	34 (14.2)	0.24
**Premedication**			
Antihypertensive drugs, *n* (%) *	38 (33)	126 (52.7)	0.0005
Antiobstructive drugs, *n* (%) *	1 (9)	4 (1.7)	0.55
Antidiabetic drugs, *n* (%) *	15 (13)	21 (8.8)	0.22
Antiplatelet agents, *n* (%) *	11 (9.6)	39 (16.3)	0.09
New oral anticoagulants, *n* (%) *	5 (4.3)	8 (3.3)	0.64
Vitamin K antagonist, *n* (%) *	20 (17.4)	53 (22.2)	0.3
**Cardiopulmonary parameter**			
Norepinephrine application rate, µg/kg/min, mean (±SD) **	0.04 (0.05)	0.02 (0.04)	0.004
Requiring norepinephrine, *n* (%) **	58 (50.4)	95 (39.7)	0.06
Systolic blood pressure, mmHg, median (IQR) **	138 (127–145)	137 (129–146)	0.66
Heart rate, beats per minute, median (IQR) **	75 (63–87)	75 (64–87)	0.86
Inspiratory oxygen fraction, mean (±SD) **	37 (12.2)	33.7 (13.8)	0.03
Endotracheal intubation, *n* (%) **	82 (71.3)	129 (54)	0.002
PEEP level, median (IQR) **	7 (6–9)	8 (6–9.8)	0.71
Arterial oxygen partial pressure (mmHg), median (IQR) **	109 (99–126)	108 (98–123)	0.13
Body temperature, centigrade, median (IQR) *	36.3 (35.5–36.8)	36.3 (35.4–37)	0.33
**Serum biomarkers**			
White blood cells, giga/L, mean (±SD) *	12.1 (5)	10.4 (4)	0.001
Hemoglobin, g/dL, mean (±SD) *	13.5 (2)	12.9 (2)	0.003
Hematocrit, %, mean (±SD) *	0.39 (0.05)	0.38 (0.05)	0.02
Cholinesterase, U/L, mean (±SD) *	8151.9 (2421)	7644.6 (2198.6)	0.05
Blood glucose, mg/dL, mean (±SD) *	192.3 (72.8)	150 (45.7)	<0.0001
Troponin I (*n* = 177), µg/dL, mean (±SD) *	0.56 (4.1)	0.27 (0.4)	0.3
Elevated Troponin I, *n* (%) *^,a^	21 (18.3)	28 (11.7)	0.72
Cortisol, µg/dL, mean (±SD) *	29.7 (20.9)	26.9 (18.6)	0.24
C-reactive protein, mg/L, mean (±SD) *	19.8 (39.5)	23.1 (39.2)	0.46
Albumin, g/L, mean (±SD) *	39.5 (5.2)	37.6 (5.4)	0.002
Creatinine, mg/dL, mean (±SD) *	0.9 (0.5)	1 (0.7)	0.39
Prothrombin time, %, mean (±SD) *	83.4 (26.9)	83.9 (26)	0.87
Partial thromboplastin time, seconds, mean (±SD) *	33.7 (15.8)	31.9 (8.6)	0.17
Antithrombin III, %/NORM, mean (±SD) *	87.7 (16.8)	89.3 (15.4)	0.5
Fibrinogen, g/L, mean (±SD) *	3.1 (1.1)	3.4 (1.1)	0.74
**Treatment regime**			
Medical treatment, *n* (%) ***	42 (36.5)	109 (45.6)	0.11
Additional Surgical Treatment, *n* (%) ***	73 (63.5)	130 (54.4)	
**Radiological data**			
Localization			
Supratentorial, lobar, *n* (%) *	37 (33.2)	85 (35.6)	0.53
Supratentorial, deep, *n* (%) *	56 (48.7)	124 (51.9)	0.57
Infratentorial, *n* (%) *	22 (19.1)	30 (12.6)	0.1
ICH volume, cm^3^, mean (±SD)	57.5 (44.5)	49.7 (40.9)	0.11
IVH, *n* (%) *	84 (73)	164 (68.6)	0.4
Hydrocephalus, *n* (%) *	56 (48.7)	102 (42.7)	0.29
**Intra-hospital outcome**			
mRS score, median (IQR) ****	5 (4–6)	5 (4–6)	0.06
Survivor, *n* (%)	71 (61.7)	172 (72)	0.52
Non-survivor, *n* (%)	44 (38.3)	67 (28)	
Favorable outcome, *n* (%) ****	42 (36.5)	101 (42.3)	0.3
Unfavorable outcome, *n* (%) ****	73 (63.5)	138 (57.7)	

SD: standard deviation, IQR: interquartile range, APACHE II: Acute Physiology and Chronic Health Evaluation II, PEEP: positive end expiratory pressure, ICH: intracerebral hemorrhage, IVH: intraventricular hemorrhage. * upon admission, ** within the first 24 h, *** during inpatient treatment, **** at discharge, ^a^ TNI value > 0.05 µg/L was defined as elevated according to the local laboratory parameters.

## Data Availability

The data presented in this study are available on request from the corresponding author.
